# Multiple Sclerosis and the Endogenous Opioid System

**DOI:** 10.3389/fnins.2021.741503

**Published:** 2021-09-17

**Authors:** Zoë Dworsky-Fried, Caylin I. Chadwick, Bradley J. Kerr, Anna M. W. Taylor

**Affiliations:** ^1^Department of Pharmacology, University of Alberta, Edmonton, AB, Canada; ^2^Neuroscience and Mental Health Institute, University of Alberta, Edmonton, AB, Canada; ^3^Department of Anesthesiology and Pain Medicine, University of Alberta, Edmonton, AB, Canada

**Keywords:** multiple sclerosis, opioid, inflammation, pain, affect, mood, immune system

## Abstract

Multiple sclerosis (MS) is an autoimmune disease characterized by chronic inflammation, neuronal degeneration and demyelinating lesions within the central nervous system. The mechanisms that underlie the pathogenesis and progression of MS are not fully known and current therapies have limited efficacy. Preclinical investigations using the murine experimental autoimmune encephalomyelitis (EAE) model of MS, as well as clinical observations in patients with MS, provide converging lines of evidence implicating the endogenous opioid system in the pathogenesis of this disease. In recent years, it has become increasingly clear that endogenous opioid peptides, binding μ- (MOR), κ- (KOR) and δ-opioid receptors (DOR), function as immunomodulatory molecules within both the immune and nervous systems. The endogenous opioid system is also well known to play a role in the development of chronic pain and negative affect, both of which are common comorbidities in MS. As such, dysregulation of the opioid system may be a mechanism that contributes to the pathogenesis of MS and associated symptoms. Here, we review the evidence for a connection between the endogenous opioid system and MS. We further explore the mechanisms by which opioidergic signaling might contribute to the pathophysiology and symptomatology of MS.

## Introduction

Multiple sclerosis (MS) is a neuroinflammatory disease characterized by chronic inflammation, demyelinating lesions, and neurodegeneration within the central nervous system (CNS) (Compston and Coles, 2008; Polman et al., 2011). MS is a highly prevalent chronic condition and a leading cause of disability in North America (Browne et al., 2014). Despite decades of research, the complex pathogenesis of MS remains incompletely understood. While its exact etiology is unknown, it is generally believed that symptoms of MS result from damage to the myelin sheath and interruption of myelinated tracts in the CNS. As such, the diagnosis of MS is limited to the recurrent presentation of clinical symptoms that indicate CNS demyelination or the identification of radiologically observable demyelinated lesions within the CNS (Karussis, 2014). More recently, the presence of oligoclonal immunoglobulin bands specifically within the cerebrospinal fluid has been offered as an alternative diagnostic criterion to a secondary clinical or radiological event (Link and Huang, 2006; Carroll, 2018). Given that different neuroanatomical locations within the CNS can be involved in disease pathophysiology, MS can present with a wide range of symptoms. Clinical symptoms of the disease include motor, cognitive, sensory, and autonomic disturbances in most patients with MS. These can manifest as loss of coordination and balance, deficits in executive functioning, vision impairment, chronic pain, and mood disorders (Compston and Coles, 2008).

There is currently no cure for MS, although various forms of pharmaceutical and rehabilitation therapies are available for treating acute attacks, improving symptoms, and modifying the disease course (Gilmour et al., 2018). Given the chronic and heterogeneous nature of the disease, treatment with multiple concurrent therapies is frequent in clinical practice. Disease-modifying therapies interfere with the course of MS through modulation or suppression of the immune system. The disease-modifying therapies that are widely used in the clinic primarily inhibit lymphocyte access to the CNS, sequester lymphocytes in primary lymphoid organs, or deplete B cells (Vargas and Tyor, 2017; Greenfield and Hauser, 2018; Hauser and Cree, 2020). However, many of these therapies have considerable undesirable side effects and confer only partial protection against disease progression and symptomatology. Thus, there is an unmet clinical need to understand the complex pathophysiology of MS and identify novel drug targets.

A growing body of evidence suggests that the opioid system may contribute to the pathogenesis of MS and the development of comorbid symptoms. Endogenous opioid signaling is seemingly altered in people with MS (Gironi et al., 2000, 2008; Ludwig et al., 2017) and treatment with opioid therapies in the clinic is largely ineffective for pain management (Kalman et al., 2002). The role of endogenous opioid peptides and their receptors in the modulation of the immune system, nociceptive processes, and mood states has been well characterized. The interaction between these systems is complex and likely contributes to the MS disease course. The goal of this review is to discuss the role of the endogenous opioid system in MS. It will highlight pathophysiological mechanisms by which dysregulated opioid signaling may contribute to MS progression and symptomatology, with a focus on pain and affective disorders.

## The Endogenous Opioid System: An Overview

The endogenous opioid system plays a critical role in modulating nociception, affective states, motivational and mood processes, neuroendocrine function, respiratory activity, and autonomic stress and immunological responses. Opioid receptors and their ligands are widely distributed throughout the central and peripheral nervous systems, the immune system, and the gastrointestinal tract. Human (Kuhar et al., 1973; Peckys and Landwehrmeyer, 1999; Peng et al., 2012) and rodent studies (Mansour et al., 1987, 1994) have characterized the widespread but distinct expression of the opioid subsystems in various tissues and cell types. In the CNS, the opioid system is classically implicated in pain signaling and antinociception. Opioid receptors are highly expressed at all levels of the central pain control network. Activation of opioid receptors within the descending pain modulatory system, which consists of the periaqueductal gray, rostral ventromedial medulla, and dorsal horn of the spinal cord (Basbaum and Fields, 1984), suppresses spinal cord nociceptive transmission and contributes to opioid-induced antinociception (Tortorici et al., 2001; Wang and Wessendorf, 2002; Lueptow et al., 2018; Wang et al., 2018a). Opioid receptors modulate a diverse range of additional functions, such as mood and the stress response, which can be attributed to their expression throughout cortical, limbic and midbrain structures (Mansour et al., 1987; Likhtik et al., 2008; Peng et al., 2012; Van’t Veer and Carlezon, 2013; Blaesse et al., 2015). Opioid receptors and their ligands are also found in neuronal and non-neuronal tissues, including cells of the immune (Wybran et al., 1979; Chuang et al., 1995; Bidlack, 2000) and enteric systems (Bagnol et al., 1997; Wood and Galligan, 2004; Poole et al., 2011).

The opioid system is comprised of three genetically distinct families of endogenous opioid peptides, including β-endorphin (derived from the precursor pro-opiomelanocortin), dynorphins (derived from pre-prodynorphin), and methionine (met)- and leucine (leu)-enkephalins (derived from pre-proenkephalin). All opioid peptides have a conserved NH2-terminal Tyr-Gly-Gly-Phe signature sequence that interacts with the classical opioid receptors: μ- (MOR), κ- (KOR), and δ-opioid receptors (DOR). Each receptor is encoded by a unique gene (*OPRM1, OPRK1, OPRD1*, respectively). The opioid receptors are all seven-transmembrane spanning proteins that couple to inhibitory G proteins (Simon, 1991; Al-Hasani and Bruchas, 2011; Benarroch, 2012) to modulate intracellular signaling cascades involving the cyclic adenosine monophosphate pathway (Vigano et al., 2003). In general, β-endorphin binds to MOR and DOR, dynorphin preferentially binds KOR, and met- and leu-enkephalin bind DOR and MOR (Benarroch, 2012). Additional opioid peptides, such as endomorphin and nociceptin/orphanin FQ (N/OFQ), which have respective affinities for MOR and nociceptin/orphanin FQ receptor (NOP), have also been described (Meunier, 1997; Zadina et al., 1997; Horvath, 2000).

## The Opioid System Modulates Immune Function

### Endogenous Opioids and Immunomodulation

A connection between the opioid and immune systems is well established and has been detailed in several excellent reviews (Salzet et al., 2000; Vallejo et al., 2004; Al-Hashimi et al., 2013; Liang et al., 2016; Plein and Rittner, 2018; Eisenstein, 2019). The immunomodulatory properties of opioids were identified over 30 years ago, when Wybran and colleagues first reported the presence of opioid receptors in normal human T lymphocytes (Wybran et al., 1979). Subsequent studies detected the presence of transcripts for all three opioid receptor subtypes (MOR, DOR, and KOR) in cells of the immune system, including T cells, B cells, and macrophages (Chuang et al., 1995; Wick et al., 1996; Sharp et al., 1997; Bidlack, 2000; Ninkovic and Roy, 2013). Several immune cell types can stimulate the release or enhance the synthesis of endogenous opioid peptides. For example, the mRNA for β-endorphin, its precursor pro-opiomelanocortin, and proenkephalin are expressed by macrophages, monocytes, granulocytes, and T and B lymphocytes(Mousa et al., 2004; Pomorska et al., 2014). Under pathological pain and inflammatory conditions, leukocytes actively synthesize and secrete opioid peptides that interact with opioid receptors within inflamed tissue to produce analgesia (Stein et al., 1990; Przewlocki et al., 1992; Cabot et al., 1997). In addition, leukocyte-derived opioid peptides suppress neuropathy-induced mechanical allodynia in mice *via* opioid receptors expressed in nociceptors at the site of nerve injury (Labuz et al., 2009).

Endomorphin 1 and 2, two endogenous opioid peptides with high specificity and affinity for MOR, were originally detected in the CNS (Zadina et al., 1997) and later identified in cells and tissues of the immune system (Jessop et al., 2000; Mousa et al., 2002; Labuz et al., 2006). Accumulating evidence suggests that endomorphins, particularly endomorphin-1, possess potent antinociceptive and anti-inflammatory properties (Przewłocka et al., 1999; Jessop et al., 2010; Zhang et al., 2018). Endomorphin-1 increases the secretion of the anti-inflammatory cytokine interleukin (IL)-10 and suppresses the secretion of proinflammatory cytokines IL-12 and IL-23 in lipopolysaccharide-activated dendritic cells *in vitro* (Li et al., 2009). Investigations involving *in vivo* rodent models of acute inflammation have shown that local or intrathecal administration of endomorphin-1 improves peripheral inflammatory pain and reduces a localized inflammatory response (Khalil et al., 1999; McDougall et al., 2004; Zhang et al., 2018). Furthermore, the addition of endomorphin-2 *in vitro* inhibits the release of inflammatory mediators, such as tumor necrosis factor (TNF)-α and IL-12 by stimulated macrophage cells (Azuma and Ohura, 2002). Endomorphin-2 also attenuates macrophage chemotaxis and phagocytosis, suggesting that this peptide alters macrophage functions related to innate host defense (Azuma and Ohura, 2002).

β-endorphin and met-enkephalin have received significant attention for their influence on T lymphocyte function. The effects of these peptides on T lymphocytes have been explored by numerous investigators with conflicting results. Early *in vitro* investigations report that β-endorphin modifies T lymphocyte function by either enhancing (Gilman et al., 1982; Gilmore and Weiner, 1989; Hemmick and Bidlack, 1990; Van Den Bergh et al., 1991; Navolotskaya et al., 2002) or inhibiting proliferation and cytokine secretion (Hough et al., 1990; Garcia et al., 1992; Marchini et al., 1995; Panerai et al., 1995). More recently, it has been demonstrated that β-endorphin suppresses IL-2 transcription (Börner et al., 2009) and potentiates IL-4 expression in a human T lymphocyte cell line (Börner et al., 2013). Met-enkephalin is implicated in the regulation of neural and non-neural cell proliferation (Zagon and McLaughlin, 1991; Zagon et al., 2002; Donahue et al., 2009). In a similar manner to β-endorphin, treatment with met-enkephalin has been shown to increase (Hucklebridge et al., 1989; Bajpai et al., 1995; Kowalski, 1998; Zagon et al., 2011; Hua et al., 2012), suppress (Brown and Van Epps, 1985; Ye et al., 1989; Ohmori et al., 2009) or have no overall effect on T lymphocyte activity or proliferation (Gilman et al., 1982; Ye et al., 1989; Kamphuis et al., 1998). Dose-dependent effects of β-endorphin (Van Den Bergh et al., 1993) and met-enkephalin (Fóris et al., 1986; Piva et al., 2005) on T lymphocyte function have been reported, which may account for inconsistencies in the literature. For instance, Piva et al. (2005) found that low doses of met-enkephalin and its metabolic derivatives stimulated the production of several cytokines by splenocytes *in vitro*, whereas higher doses were suppressive (Piva et al., 2005). The discrepancies between investigations may also be due to differences in methodologies, including the concentration of the peptide in question, whether the peptides were natural or synthetic, whether the cells were stimulated or homeostatic, the presence or absence of serum in culture, and the types of assays used to assess cell proliferation. Nevertheless, it is clear that endogenous opioid peptides can influence immune cell function and may therefore contribute to immune system pathology as seen in MS.

### Clinical Use of Opioids and Immunomodulation

Preclinical and clinical studies have demonstrated that exogenously administered opioids exert robust immunomodulatory effects, which are highly dependent on the type of opioid and the duration of exposure (Sacerdote et al., 2000; Martucci et al., 2004; Al-Hashimi et al., 2013; Franchi et al., 2019). For instance, chronic morphine treatment appears to have potent modulatory effects on the immune system, whereas codeine and hydromorphone do not (Sacerdote et al., 1997; Ninkovic and Roy, 2013). The modulatory effects of clinically used opioids on peripheral immune cells have been most extensively studied *in vitro* and *in vivo* (Vallejo et al., 2004; Ninkovic and Roy, 2013). The majority of experiments that involved the *in vivo* administration of opiates, such as morphine and heroin, or the addition of MOR, KOR, and DOR agonists to cell cultures *in vitro*, indicate significant suppression of the immune system. Immunosuppression was reported as reduced natural killer cell activity (Shavit et al., 1986b, a; Weber and Pert, 1989; Yeager et al., 1995; Sacerdote et al., 1997; Gavériaux-Ruff et al., 1998), cytokine and chemokine production by peripheral blood mononuclear cells (Bussiere et al., 1993; Chao et al., 1993; Bonnet et al., 2008) and monocytes (Bussiere et al., 1993; Bian et al., 1995; Roy et al., 1998), T and B cell reactivity (Sacerdote et al., 1997; Govitrapong et al., 1998), phagocytic activity (Tubaro et al., 1985; Casellas et al., 1991; Rojavin et al., 1993; Szabo et al., 1993; Tomassini et al., 2004), as well the induction of macrophage apoptosis (Bhat et al., 2004; Lin et al., 2021). Additional evidence supporting the immunosuppressive role of opioid analgesics emerges from epidemiological studies showing increased prevalence of infections such as HIV, pneumonia, hepatitis and tuberculosis among opioid users (Nath et al., 2002; Quaglio et al., 2002; Roy et al., 2011; Wiese et al., 2018).

Multiple sclerosis immunopathology is generally thought to be mediated by myelin-reactive CD4^+^ T helper (Th) cells. Autoreactive effector CD4^+^ T cells can differentiate into Th1 or Th2 effector cells based upon their functions and cytokine profile. Disruption to the Th cell balance, especially the decrement of the Th1/Th2 ratio, is implicated in the development of several autoimmune diseases, including MS (van Langelaar et al., 2018; Kunkl et al., 2020). Similarly, several studies indicate that Th17 cells, a subset of CD4^+^ T-cells that produces IL-17, play a key role in the pathogenesis of various inflammatory and autoimmune diseases (Waite and Skokos, 2012; Yasuda et al., 2019; Moser et al., 2020). Modulation of T cell differentiation and function by opioids has been well documented and may therefore be relevant in MS immunopathology. Morphine has been shown to selectively direct T cells toward Th2 differentiation *in vitro* and *in vivo*, resulting in a shift in the Th1/Th2 balance (Roy et al., 2004; Han et al., 2020). Gao et al. (2012) demonstrated that the effects of morphine on CD4^+^ T lymphocytes isolated from healthy volunteers include altered cytokine expression, suppression of T cell apoptosis and Th cell differentiation, as well as an imbalance in the ratio of Th1/Th2 cells (Gao et al., 2012). Morphine dose-dependently suppresses the proliferative activity of phytohemagglutinin-stimulated T lymphocytes isolated from opioid-naive subjects *in vitro* (Govitrapong et al., 1998). In line with these findings, heroin users show reduced CD4^+^ T cell proliferative activity upon stimulation *in vitro* and an altered Th1/Th2 balance when compared with healthy controls and individuals on opioid maintenance therapy (Sacerdote et al., 2008; Riß et al., 2012). In rats, acute morphine exposure (Peng et al., 2020) and moderate doses of naltrexone (Xu et al., 2020) have been shown to suppress Th17 cell expression and function, as well as disrupt the balance between Th1 and Th2 cells (Xu et al., 2020). Moreover, treatment with chronic morphine enhances Th17 cell functional activity in peripheral blood mononuclear cells isolated from non-human primates (Cornwell et al., 2013).

Glial cells, consisting primarily of microglia, astrocytes, and oligodendrocytes, represent immune cells of the CNS. Several laboratories have demonstrated that microglia and astrocytes become activated in response to chronic morphine exposure, inducing the upregulation of proinflammatory cytokines IL-1, IL-6, and TNF-α, microglial and astrocytic activation markers, and purinergic receptors P2×4 and P2×7 (Raghavendra et al., 2002; Tawfik et al., 2005; Cui et al., 2006; Horvath and Deleo, 2009; Hutchinson et al., 2009; Watkins et al., 2009). Interfering with glial function reduces opioid tolerance and opioid-induced hyperalgesia, providing further evidence for the modulatory role of opioids on glial cells (Raghavendra et al., 2002, 2003; Eidson and Murphy, 2013).

## Dysregulation of the Opioid System in Multiple Sclerosis

Overwhelming evidence indicates that endogenous opioid peptides and clinically used opioids have significant influence on innate and adaptive immunity. While the etiology of MS remains incompletely understood, it is recognized that the pathogenesis and progression of this disease are mediated by the immune system. Thus, it is important to elucidate the relationship between the opioid and immune systems in the context of MS to gain mechanistic insight into pathophysiological processes associated with this disease.

### The Role of the Opioid System in the Pathogenesis and Progression of Multiple Sclerosis

#### Disease-Related Changes in Endogenous Opioid Peptide Concentrations

Human and animal studies provide converging lines of evidence indicating that perturbations to the endogenous opioid system contribute to the pathogenesis of several autoimmune disorders, including MS. Patients with MS show decreased concentrations of endogenous opioid peptides β-endorphin and enkephalin in peripheral blood mononuclear cells and cerebrospinal fluid samples compared with healthy controls (Panerai et al., 1994; Gironi et al., 2000, 2008; Ludwig et al., 2017). Mice with experimental autoimmune encephalomyelitis (EAE), the most commonly used preclinical murine model of MS, also show a marked reduction in serum concentrations of met-enkephalin compared with baseline levels and with controls prior to the onset of clinical behavioral signs of disease (Ludwig et al., 2017; Patel et al., 2020). Studies assessing changes in endogenous opioid peptide and receptor expression in MS patients and animal models are summarized in Table 1. EAE is a CD4^+^ T lymphocyte-mediated demyelinating autoimmune disease of the CNS, characterized by widespread central inflammation and infiltration of T cells and monocytes into the CNS (Robinson et al., 2014). The EAE model shares many pathological features with MS, including neuroinflammation, demyelination, neurodegeneration, axonopathy and pain (Olechowski et al., 2009; Kipp et al., 2012; Potter et al., 2016; Catuneanu et al., 2019). Given the role of met-enkephalin in modulating adaptive immune cell reactivity (Zagon and McLaughlin, 1991; Zagon et al., 2002; Malendowicz et al., 2005; Donahue et al., 2009), reduced serum enkephalin levels in MS patients may promote immune cell proliferation and drive immune-mediated flares. Indeed, a series of investigations reveal that increasing levels of met-enkephalin confer a neuroprotective effect in mice with EAE and people with MS (Gironi et al., 2008; Zagon et al., 2009, 2010; Ludwig et al., 2017). Jankovic and Maric (1987) demonstrated that injections of met-enkephalin to rats with EAE prevents or delays paralysis. Daily administration of met-enkephalin to mice with EAE at the time of disease induction prevents the onset and progression of disease, and decreases overall disease severity, areas of demyelination, and activated glia in the spinal cord relative to saline-treated controls (Zagon et al., 2010; Rahn et al., 2011; Patel et al., 2020). In mice with established EAE, treatment with met-enkephalin halts the progression of disease, improves the clinical behavioral scores, and reduces the number of activated glia, T cells, and demyelinated areas in the spinal cord (Campbell et al., 2012, 2013). Moreover, treatment with met-enkephalin in mice with relapse-remitting EAE results in less severe clinical disease scores, fewer and shorter relapses, and diminished glial activation and spinal cord pathology compared to controls (Hammer et al., 2013). In summary, results from studies involving exogenous therapy with enkephalins in the EAE model indicate beneficial effects of modulating endogenous opioid levels and suggest that the opioid system plays an integral role in the underlying disease process.

**TABLE 1:** Changes in endogenous opioid peptide and receptor expression in MS patients and animal models.

Opioid peptide/receptor	Disease	Expression change	References
Met-enkephalin	MS (human)	↓ protein in serum	Ludwig et al., 2017
	EAE (mouse)	↓ protein in serum	Ludwig et al., 2017; Patel et al., 2020
β-endorphin	MS (human)	No change in serum	Ludwig et al., 2017
	MS (human)	↓ protein in peripheral blood mononuclear cells	Gironi et al., 2000
	EAE (mouse)	No change in serum	Ludwig et al., 2017
MOR, KOR, and DOR	TMEV (mouse)	↓ mRNA in spinal cord	Lynch et al., 2008

#### Low Dose Naltrexone Therapy

Naltrexone is a non-selective opioid receptor antagonist that is primarily prescribed for the treatment of opioid addiction in daily doses of at least 50 mg (Minozzi et al., 2011). When prescribed at the lowest dosage levels (1–4.5 mg), it acts as an immune modulator by reducing the inflammatory glial response (Mattioli et al., 2010; Younger et al., 2014), in addition to systemically upregulating endogenous opioid signaling by transient opioid receptor blockade (Gironi et al., 2008; Ludwig et al., 2017). Treatment with low dose naltrexone has been demonstrated to improve symptoms in a variety of chronic inflammatory conditions, including Crohn’s disease (Lie et al., 2018), fibromyalgia (Younger and Mackey, 2009; Younger et al., 2013), complex regional pain syndrome (Chopra and Cooper, 2013; Weinstock et al., 2016), and MS (Gironi et al., 2008; Cree et al., 2010; Sharafaddinzadeh et al., 2010; Ludwig et al., 2017).

Preclinical studies wherein mice were immunized with EAE report beneficial effects of low dose naltrexone treatment in modulating disease processes (Zagon et al., 2009; Hammer et al., 2013, 2015; Ludwig et al., 2017). Therapy with low dose naltrexone, but not high dose naltrexone, prevents neurological signs of disease, suppresses disease onset and progression, and reduces the number of activated astrocytes in the spinal cord of EAE mice (Zagon et al., 2009). In addition, mice with chronic EAE receiving low dose naltrexone show reduced sensitivity to heat relative to saline-treated EAE mice (Ludwig et al., 2017). In studies with mice immunized with relapsing-remitting EAE, treatment with low dose naltrexone initiated at the time of established disease significantly diminishes behavioral scores and increases the incidence and lengthens the time of remissions compared with EAE mice treated with saline (Hammer et al., 2015). Low dose naltrexone therapy also reduces numbers of inflammatory cells, such as microglia, CD3^+^ T cells, and activated astrocytes, as well as areas of demyelination in the lumbar spinal cord (Hammer et al., 2015). Recent work extended these findings and demonstrated that treatment with low dose naltrexone preserves myelin basic protein expression and the number of oligodendrocytes within the spinal cord in EAE mice relative to control animals (Patel et al., 2020).

Results from clinical trials suggest that low dose naltrexone treatment enhances the quality of life of patients with MS (Gironi et al., 2008; Cree et al., 2010) and that treatment is well tolerated (Gironi et al., 2008; Cree et al., 2010; Sharafaddinzadeh et al., 2010). The first multi-center open-label pilot study involving 40 patients with primary progressive MS reported that spasticity was significantly reduced following 6 months of treatment with low dose naltrexone (Gironi et al., 2008). Levels of β-endorphin in patients’ peripheral blood mononuclear cells increased concurrently with low dose naltrexone administration, providing support for a potential mechanism of action. The interpretation of these results, however, is limited by the uncontrolled design of the study and the small sample size. More recently, low dose naltrexone therapy was shown to restore depressed serum enkephalin levels of MS patients to non-MS patient concentrations (Ludwig et al., 2017). An additional randomized, placebo-controlled study comprised of 60 MS patients found that 8 weeks of therapy with low dose naltrexone was associated with significant improvement in self-reported mental health outcome measures (Cree et al., 2010). By contrast, a 17-week randomized, double-blind, placebo-controlled clinical trial involving 96 MS patients found no statistically significant improvements in self-reported quality of life following low dose naltrexone treatment between groups (Sharafaddinzadeh et al., 2010). The authors noted that low dose naltrexone therapy was relatively safe and that longer trials are needed to conclude that there is no beneficial effect.

Nevertheless, high quality clinical studies evaluating the therapeutic effects of low dose naltrexone in treating MS are lacking. Of the completed studies, results indicate that low dose naltrexone is generally safe, compatible with currently recommended MS treatments and well tolerated, but do not show significant changes in symptoms beyond quality-of-life improvements. Low dose naltrexone may be a promising alternative or adjunct therapy for MS treatment; however, additional research is necessary to determine the clinical potential of low dose naltrexone use in MS.

#### The Kappa Opioid System as a Potential Therapeutic Target

There is emerging evidence indicating that targeting the kappa opioid system may be a promising therapeutic target for attenuating the progression of MS *via* remyelination (Wang and Mei, 2019). An *in vitro* myelination assay has shown that KOR agonism promotes differentiation of oligodendrocyte precursor cells (OPCs) into mature oligodendrocytes and subsequent myelination (Mei et al., 2016). This beneficial effect on myelination is abolished in mice that have KOR conditionally knocked out in OPCs. This study provides support that KOR ligands are directly acting on KORs expressed on OPCs and suggests that future studies should consider targeting this receptor for remyelination therapy (Mei et al., 2016). In line with these results, treatment with a selective KOR agonist, U50,488H, has been shown to enhance remyelination in lysolecithin-, hypoxia-, and cuprizone-induced demyelination models (Mei et al., 2016; Wang et al., 2018b), and has since been replicated in the EAE model using a delayed treatment schedule (Denny et al., 2021). Furthermore, Du et al. (2016) found that administration of U50,488H reduced the severity of motor impairments in EAE mice through promoting oligodendrocyte differentiation and remyelination. The authors also induced EAE in opioid receptor knockout mice. MOR-deficient mice did not show any changes in the severity or progression of EAE, DOR knockout mice only displayed a small increase in peak disease severity, and genetic deletion of KOR worsened disease severity compared with wild-type (Du et al., 2016). These data indicate that KOR contributes to remyelinating processes and that targeting the kappa opioid system is an intriguing avenue for developing novel therapeutics for the treatment of MS.

### The Endogenous Opioid System and Multiple Sclerosis Symptomatology: A Focus on Pain and Affect

#### The Opioid System and Pain

Chronic pain is one of the most frequent and debilitating symptoms of MS, affecting between 50–80% of patients over the course of their disease (Österberg et al., 2005). MS-related pain is characterized by hyperalgesia (enhanced pain responses to noxious input) and allodynia (perception of innocuous stimuli as painful). MS patients often describe their pain as constant, bilateral aching, burning, and pricking sensations in both the lower and upper extremities (Österberg et al., 2005). Classical pain treatments, such as opioid therapy, are typically ineffective in treating MS-related pain, with only a minority of patients receiving significant relief (Kalman et al., 2002). Chronic pain associated with MS represents a significant clinical and societal burden. As the general population ages, it can be expected that the rates of MS will only increase, thus it is becoming increasingly imperative that adequate treatments for pain in this disease are developed. A more thorough understanding of the basic mechanisms driving pain in MS is necessary for the development of novel therapies to improve pain management for this patient population.

There are a large number of autoimmune and inflammatory diseases with different etiologies and symptomatologies, including rheumatoid arthritis, irritable bowel syndrome, complex regional pain syndrome, and MS, and pain appears to be a common factor in most of these conditions (Mifflin and Kerr, 2017). Activation of the endogenous opioid system is evidenced in a variety of these conditions that are associated with the development of pathological pain. Human positron emission tomography studies show that compared with controls, patients with rheumatoid arthritis (Jones et al., 1994), complex regional pain syndrome (Klega et al., 2010), and central neuropathic pain following stroke (Willoch et al., 2004) have reduced opioid receptor binding potential at several neural loci involved in the central pain matrix and emotional regulation. This may indicate increased occupancy of receptors by endogenous opioid peptides or a reduction in available receptors for binding. Work from animal studies further corroborate the release of endogenous peptides in chronic pain states. For example, experimental hindpaw inflammation induces a rapid increase in pre-prodynorphin mRNA and a prolonged increase in a dynorphin peptide in the spinal cord (Iadarola et al., 1988). These data collectively suggest that several inflammatory pain states are associated with the release and binding of endogenous opioids to their cognate receptors. However, studies that directly investigate the contribution of the opioid system to pain hypersensitivity in MS and EAE are limited.

As discussed above, the role of endogenous opioids in MS has primarily been evaluated in the context of immunity and disease progression. Although the contribution of opioidergic neurotransmission to MS-related pain remains relatively unexplored, there is evidence to indicate that dysfunction of the opioid system may be implicated in the development and maintenance of pain in this disease (summarized in Table 2). Similar to that observed in other chronic pain conditions (Arnér and Meyerson, 1988; Zurek et al., 2001; Luger et al., 2002; Rowbotham et al., 2003; Chen et al., 2013; Kissin, 2013), opioid analgesics often provide inadequate relief for MS patients, except at high doses that might enhance the risk for adverse side effects (Kalman et al., 2002). Animal models of MS-related pain also show reduced opioid analgesia compared with controls (Lynch et al., 2008; Dworsky-Fried et al., 2021). We previously reported that morphine lacks potent analgesic efficacy in female mice induced with EAE at a time point that was associated with peak pain hypersensitivity and inflammation in the brain (Dworsky-Fried et al., 2021). Consistent with these findings, male and female mice infected with Theiler’s murine encephalomyelitis virus (TMEV) as a model for MS display a loss of morphine analgesia compared to uninfected control mice (Lynch et al., 2008). Mice infected with TMEV also show reductions in spinal cord mRNA levels of all three opioid receptors (MOR, DOR, and KOR), which correlated with the development of thermal and mechanical hyperalgesia (Lynch et al., 2008). While this study did not investigate the causal relations between receptor changes and pain behaviors, decreases in spinal opioid receptors may explain the increased central neuropathic pain commonly observed in MS patients (O’Connor et al., 2008; Khan and Smith, 2014). From a clinical perspective, dysregulation of the opioid system might also help to explain the poor patient response to this class of analgesics (Kalman et al., 2002). Taken together, these findings provide support for the hypothesis that loss of endogenous antinociceptive processes mediated by the opioid system contribute to MS- and EAE-related pain. As such, restoring opioid system function may be a viable target for novel analgesic drugs and therapeutics to manage pain in this disease. Additional investigations are needed to understand the contribution of endogenous opioids and receptors to pathological pain in MS.

**TABLE 2:** Effects of exogenous opioid treatment in MS patients and animal models.

Opioid receptor ligand	Disease	Main findings	References
Low dose naltrexone	EAE (mouse)	↓ motor impairment	Rahn et al., 2011; McLaughlin et al., 2015; Patel et al., 2020
		↓ activated astrocytes in spinal cord; ↓ incidence of EAE	Rahn et al., 2011
		↓ T and B splenocytes at disease onset	McLaughlin et al., 2015
		↑ CD4^+^ T cells in CNS; ↓ CD3^+^ and CD4^+^ T cells in spinal cord	Hammer et al., 2016
		↑ met-enkephalin protein in serum; ↓ leukocytes and eosinophils in blood; ↓ heat sensitivity (hot plate)	Ludwig et al., 2017
		↑ myelin basic protein in spinal cord; ↑ oligodendrocytes in spinal cord	Patel et al., 2020
	MS (human)	↑β-endorphin in peripheral blood mononuclear cells; ↓ spasticity; ↑ pain	Gironi et al., 2008
		No change in quality of life	Sharafaddinzadeh et al., 2010
		↑ quality of life; ↑ mental health; ↓ pain	Cree et al., 2010
		↑ met-enkephalin protein in serum	Ludwig et al., 2017
Met-enkephalin	EAE (rat)	↓ lesions in CNS; ↓ incidence of EAE	Jankovic and Maric, 1987
	EAE (mouse)	↓ motor impairment	Zagon et al., 2010; Rahn et al., 2011; Campbell et al., 2012; Hammer et al., 2013, 2015; McLaughlin et al., 2015; Patel et al., 2020
		↓ activated astrocytes in spinal cord	Zagon et al., 2010; Rahn et al., 2011; Campbell et al., 2013; Hammer et al., 2013, 2015
		↓ damaged neurons in spinal cord	Zagon et al., 2010; Campbell et al., 2012; Hammer et al., 2013, 2015
		↓ incidence of EAE; ↑ disease remission	Zagon et al., 2010; Rahn et al., 2011
		↓ CD3^+^ T cells in spinal cord	Campbell et al., 2012; Hammer et al., 2013, 2015, 2016
		↓ demyelination in spinal cord; ↓ astrocyte proliferation; ↓ microglia/macrophages in spinal cord	Campbell et al., 2012; Hammer et al., 2013, 2015
	EAE (mouse)	↓ heat sensitivity (hot plate)	Campbell et al., 2012
		↓ number of relapses; ↓ time in relapse	Hammer et al., 2013, 2015
		↓ T and B cells at onset; ↑ Th1 and Th17 cells in spinal cord	McLaughlin et al., 2015
		↓ CD4^+^ T cells in CNS	Hammer et al., 2016
		↑ myelin basic protein in spinal cord; ↑ oligodendrocytes in spinal cord	Patel et al., 2020
U50,488H (KOR agonist)	EAE (mouse)	↓ spinal demyelination; ↑ spinal myelin thickness; ↓ motor impairment	Du et al., 2016
	EAE (mouse)	↓ motor impairment; ↑ incidence of remission; ↑ time in remission	Denny et al., 2021
Quinoxaline-derivatives (KOR agonist)	EAE (mouse)	↓ motor impairment; ↓ B cells in CNS; ↓ Th17 cells in CNS; ↑ Treg cells in CNS	Tangherlini et al., 2019
Nalfurafine (KOR agonist)	EAE (mouse)	↓ motor impairment; ↓ B cells in CNS; ↓ CD8^+^ T cells in CNS; ↑ myelinated axons; ↑ myelin thickness; ↑ incidence of remission; ↑ time in remission; ↓ number of relapses	Denny et al., 2021

#### The Opioid System and Negative Affect

Mental health comorbidities are highly prevalent among individuals with MS. Depression is the most common of these comorbidities, affecting approximately 50% of people with MS (Marrie et al., 2009). This is nearly three times higher than the current rate of depression in the general United States population (Villarroel and Terlizzi, 2020). People living with MS are twice as likely to commit suicide as someone without MS, with suicidal ideation showing a similar increase (Turner et al., 2006; Feinstein and Pavisian, 2017). Studies using functional magnetic resonance imaging reveal that MS patients with chronic pain show structural and functional alterations in brain regions involved in reward processing, which are associated with impaired reward responsiveness and depression (Pardini et al., 2013; Seixas et al., 2016; Heitmann et al., 2020). Pharmacological treatment of depression is not often pursued in people with MS because there is limited evidence to support a beneficial effect in this patient population (Minden et al., 2014; Patten et al., 2017). In addition, there are reports of a possible harmful interaction between some antidepressants and the initiation of fingolimod use—a common immunomodulatory drug prescribed for managing MS (Patten et al., 2017). This undertreatment of negative affect in people with MS poses a significant problem and has profound impacts on quality of life. As such, understanding whether dysfunction of the opioid system is involved in the etiology of mood disorders in MS is critical for effective management of this condition.

In recent years, dysfunction of the endogenous opioid system has garnered significant attention as a key component in depressive symptomatology and pathophysiology (Hegadoren et al., 2009; Peciña et al., 2019). Preclinical and clinical studies provide evidence of opioid system involvement in negative affective states. For instance, individuals with a history of depression who commit suicide have increased MOR density in the brain, specifically in the prefrontal cortex, temporal cortex, and basal ganglia (Gross-Isseroff et al., 1990; Gabilondo et al., 1995). Women with major depressive disorder exhibit increased MOR system activation compared with control subjects (Kennedy et al., 2006). Indeed, increases in KOR and MOR protein in the blood serum have become targets for biomarker identification of major depressive disorder in humans (Al-Hakeim et al., 2019, 2020). Clinical reports have described the effectiveness of MOR agonists, including oxycodone, tramadol, oxymorphone, and buprenorphine, as well as β-endorphin, in patients suffering from depression (Darko et al., 1992; Bodkin et al., 1995; Stoll and Rueter, 1999; Shapira and DeGraw, 2001). Preclinical studies using animal paradigms of depression corroborate these findings (Rojas-Corrales et al., 1998, 2004). The evidence of opioid system dysfunction as an important mechanism driving negative affect suggests that altered opioidergic mechanisms may also play a role in the development of comorbid mood disorders in MS.

The EAE mouse model is a useful tool for modeling the affective symptoms of MS (Pollak et al., 2002). Affective disturbances, such as depressive- and anxiety-like behaviors, and cognitive and memory dysfunction have been noted early in the EAE disease course, similar to the clinical population (Pollak et al., 2002; Peruga et al., 2011; Acharjee et al., 2013; Olechowski et al., 2013). Several studies report that mice with EAE show higher levels of anxious and depressive behaviors than control mice in a variety of experimental paradigms including the elevated plus maze, open field test, forced swim test, tail suspension test, and social interaction test (Acharjee et al., 2013; Olechowski et al., 2013). Using the conditioned place preference assay to assess drug reinforcement, our group has recently demonstrated that morphine reward is blunted in mice with EAE (Dworsky-Fried et al., 2021). This finding indicates that affective and reward processing is disrupted in the EAE model, and is consistent with other reports indicating dysregulated reward processing in chronic pain states (Ozaki et al., 2002, 2003; Martin et al., 2007; Petraschka et al., 2007; Niikura et al., 2008). Although literature that focuses on the involvement of the endogenous opioid system in MS-related mood and affective disorders is limited, existing evidence warrants further exploration into this research avenue. Understanding whether disruptions to endogenous opioid signaling contribute to impaired mood and reward regulation in MS is paramount for future treatment of this comorbidity.

## Conclusion

Opioid peptides and their receptors are intimately involved in regulating various aspects of immune function, nociceptive processing, and affective states. Dysregulation of the opioid system may be an important mechanism to help explain the pathophysiology of MS, as well as the pathological pain and disordered mood commonly observed in this disease. Therefore, it is of interest to further investigate and consider the opioid system as a potentially attractive therapeutic target for MS and its symptoms.

Although MS is a highly prevalent autoimmune disorder, a comprehensive understanding of the pathogenesis and symptomatology of the disease is still lacking. Accumulating data imply functional association between endogenous opioid systems and MS. Patients with MS and animal models show decreased levels of endogenous opioid peptides compared with healthy controls (Panerai et al., 1994; Gironi et al., 2000, 2008; Ludwig et al., 2017; Patel et al., 2020) and some clinical trials have shown beneficial effects of therapies that enhance endogenous opioid concentrations (Gironi et al., 2008; Ludwig et al., 2017). Moreover, opioid analgesics often provide inadequate pain relief for patients with MS. Given the complex interactions between the opioid and immune systems, nociceptive processing, and mood regulation as discussed in this review, targeting opioidergic mechanisms may provide an effective measure to interfere with the development and progression of MS and improve disabling symptoms.

## Author Contributions

ZD-F and AT conceptualized the work. ZD-F drafted the initial manuscript and edited the text. ZD-F and CC reviewed the literature and contributed to the final manuscript text. CC created the tables. BK and AT provided the editorial comments. All authors contributed to the manuscript revision and approved the submitted version.

## Conflict of Interest

The authors declare that the research was conducted in the absence of any commercial or financial relationships that could be construed as a potential conflict of interest.

## Publisher’s Note

All claims expressed in this article are solely those of the authors and do not necessarily represent those of their affiliated organizations, or those of the publisher, the editors and the reviewers. Any product that may be evaluated in this article, or claim that may be made by its manufacturer, is not guaranteed or endorsed by the publisher.
